# Recent Advances in Carbon Nitride-Based S-scheme Photocatalysts for Solar Energy Conversion

**DOI:** 10.3390/ma16103745

**Published:** 2023-05-15

**Authors:** Yawei Xiao, Xu Tian, Yunhua Chen, Xuechun Xiao, Ting Chen, Yude Wang

**Affiliations:** 1 National Center for International Research on Photoelectric and Energy Materials, School of Materials and Energy, Yunnan University, Kunming 650091, China; 2 Department of Physics, Yunnan University, Kunming 650504, China; 3 Institute of Materials Science & Devices, School of Materials Science and Engineering, Suzhou University of Science and Technology, Suzhou 215009, China; 4 Yunnan Key Laboratory of Carbon Neutrality and Green Low-Carbon Technologies, Yunnan University, Kunming 650504, China

**Keywords:** carbon nitride, S-scheme heterojunction, electron transfer, photocatalysis, solar energy conversion

## Abstract

Energy shortages are a major challenge to the sustainable development of human society, and photocatalytic solar energy conversion is a potential way to alleviate energy problems. As a two-dimensional organic polymer semiconductor, carbon nitride is considered to be the most promising photocatalyst due to its stable properties, low cost, and suitable band structure. Unfortunately, pristine carbon nitride has low spectral utilization, easy recombination of electron holes, and insufficient hole oxidation ability. The S-scheme strategy has developed in recent years, providing a new perspective for effectively solving the above problems of carbon nitride. Therefore, this review summarizes the latest progress in enhancing the photocatalytic performance of carbon nitride via the S-scheme strategy, including the design principles, preparation methods, characterization techniques, and photocatalytic mechanisms of the carbon nitride-based S-scheme photocatalyst. In addition, the latest research progress of the S-scheme strategy based on carbon nitride in photocatalytic H_2_ evolution and CO_2_ reduction is also reviewed. Finally, some concluding remarks and perspectives on the challenges and opportunities for exploring advanced nitride-based S-scheme photocatalysts are presented. This review brings the research of carbon nitride-based S-scheme strategy to the forefront and is expected to guide the development of the next-generation carbon nitride-based S-scheme photocatalysts for efficient energy conversion.

## 1. Introduction

An increasingly tight energy supply has become a major constraint on social development [1]. Since the industrial revolution, mankind has used fossil energy to achieve the rapid development of civilization, but with it comes the growing problem of energy depletion and environmental pollution [2,3]. At present, the energy used by mankind is still mainly non-renewable fossil fuels. The massive consumption of carbon-based fuels has led to an explosion of greenhouse gas emissions in the environment, causing catastrophic consequences, such as global warming, glacial melting, a rise in sea level, and an ecological imbalance [4]. Therefore, the development of clean, renewable energy is of great significance to maintain the energy supply and curb environmental pollution. Among the many types of clean energy (wind power, hydropower, solar energy, biomass, etc.), solar energy is a universal, clean, and lasting energy, and its exploitation is a potential way to alleviate energy and environmental problems [5,6,7]. Although solar energy has many advantages, its fragmentation and instability create obstacles for the effective use of this energy [8]. Developing new technologies to overcome the limitation of natural factors is the key to achieving the full use of solar energy. In 1972, Hond et al. found that a TiO_2_ photoelectrode can decompose H_2_O into H_2_ and O_2_ under UV light, and this discovery opened a new window for the exploitation of solar energy [9]. Photocatalysis technology has made breakthroughs in H_2_O splitting, CO_2_ reduction, pollutant degradation, air purification, antibacterial properties, and self-cleaning techniques after decades of development and is gradually becoming one of the important ways to solve energy and environmental problems [10,11,12,13,14,15].

**Figure 1:**
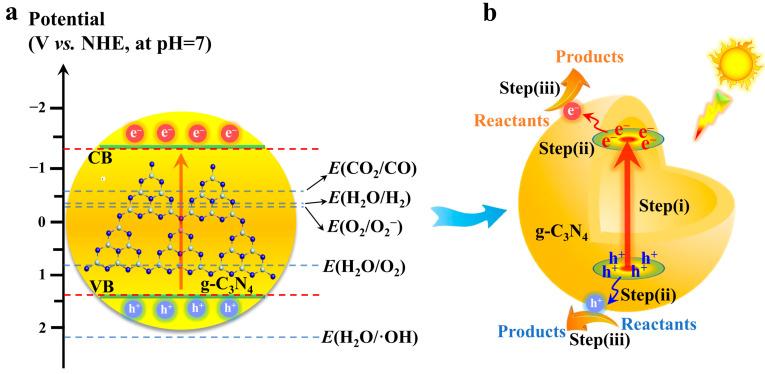
(**a**) The relative position of some representative redox potential and the band of carbon nitride, (**b**) the diagram of the main steps of the photocatalytic reaction on carbon nitride.

Photocatalysts are the key hub for solar energy conversion. The construction of high-performance photocatalysts is one of the core tasks in the development of photocatalytic technology. Metal oxides and sulfide semiconductors (TiO_2_, CdS, etc.) have been successfully used as photocatalysts [16,17,18,19,20]. However, metal oxides usually have low spectral utilization due to their wide bandgap. Metal sulfides are prone to photo-corrosion and have potential problems with metal dissolution [21,22]. Different from traditional metal-based photocatalyst, carbon nitride is an organic polymer semiconductor with a graphite-like two-dimensional layered structure. In 2009, g-C_3_N_4_ was successfully used for photocatalytic water splitting. Since then, carbon nitride has gradually become a new star in the field of photocatalysis due to its abundant raw materials, simple preparation, suitable energy band structure, and high stability [23,24,25,26]. The conduction band (CB) potential of carbon nitride is about −1.3 eV vs. NHE, which is more negative than most semiconductors. Therefore, the photogenerated electrons on carbon nitride are thermodynamically easier to drive some reactions with high reduction potentials [27]. The valence band (VB) potential of carbon nitride is about 1.4 eV vs. NHE, and its band structure is perfectly suited to photocatalytic water splitting (Figure 1a) [28]. Although carbon nitride is a promising semiconductor photocatalyst, the pristine bulk carbon nitride still suffers from a narrow spectral response region, serious electron and hole recombination, and low photocatalytic efficiency [29,30]. The photocatalytic pathway on carbon nitride can be roughly decomposed into three steps: (i) carbon nitride absorbs a photon to produce electron-hole pairs; (ii) electron-hole pairs separate and transfer to the surface of carbon nitride; and (iii) the electron-hole reacts with the reactants adsorbed on carbon nitride (Figure 1b). The photocatalytic efficiency of carbon nitride is limited by the above three factors. Similar to the cannikin law, any one of these factors may lead to the poor photocatalytic activity of carbon nitride.

In response to the shortcomings of carbon nitride, a variety of modification schemes have been tried, such as morphological control, doping, defect engineering, noble metal modification, etc. These measures can effectively optimize the charge separation, spectral absorption range, and the number of active sites of carbon nitride [31,32,33,34,35]. However, carbon nitride has a low dielectric constant and high resistivity, and its high exciton binding energy and high resistivity lead to low charge separation efficiency and poor carrier migration ability, which seriously restricts the performance of carbon nitride [36,37]. Yu et al. have likened the process of electron transitions in semiconductors to upward throwing [38]. Similar to the fall of a thrown object under gravity, electrons excited from the VB to the CB will also recombine with the holes under the action of coulomb force. The coulomb constant is greater than the universal gravitational constant, which means that it is very difficult to inhibit the recombination of the electron and hole in pure carbon nitride. The optical response range of a semiconductor is inversely proportional to the bandgap. However, the narrowing of the bandgap implies that the CB position moves down or the VB position moves up, which will lead to a significant decrease in the redox activity of carbon nitride. Therefore, it is difficult to achieve a broad spectral response and strong redox capabilities in pure carbon nitride [39,40]. Multi-component heterojunction photocatalysts offer unique advantages over single-component photocatalysts in terms of the charge separation [41,42,43,44,45]. According to the equation of the coulomb force: F_c_ = kq_e_q_h_/r^2^, heterojunction photocatalysts can realize the spatial separation of photogenerated carriers, increase the distance between electrons and holes, and reduce the coulomb force between them, thus inhibiting the recombination of photogenerated carriers [46].

**Figure 2:**
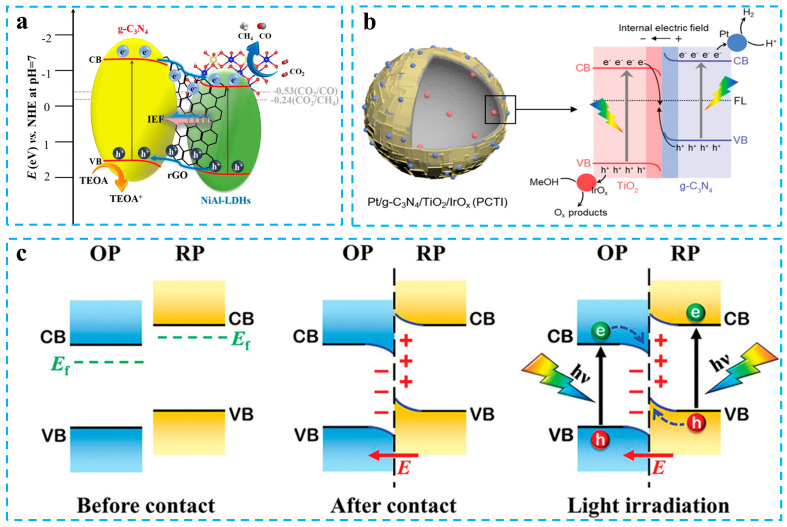
(**a**) Charge transfer in g-C_3_N_4_/rGO/NiAl-LDHs type-II heterojunction. Reprinted with permission from ref. [54], Copyright 2020 Elsevier. (**b**) Charge transfer in Pt/g-C_3_N_4_/TiO_2_/IrOx Z-scheme heterojunction. Reprinted with permission from ref. [59], Copyright 2022 Wiley. (**c**) Charge transfer in S-scheme heterojunctions. Reprinted with permission from ref. [46], Copyright 2021 Wiley.

The combination of carbon nitride with other semiconductors can realize the spatial separation of charges and bring other unexpected improvements [47,48,49,50]. The combination of carbon nitride with narrow bandgap semiconductors can increase the spectral response range, and a combination with porous semiconductors can increase the active sites [51,52,53]. The construction of type-II heterojunctions can inhibit the rapid recombination of carriers to some extent. Zhou et al. enhanced the charge separation ability of the photocatalyst by constructing a g-C_3_N_4_/rGO/NiAl-LDHs type-II heterojunction [54]. Photogenerated electrons transfer from the g-C_3_N_4_ to the CB of NiAl-LDHs, and photogenerated holes in the NiAl-LDHs migrate to the VB of g-C_3_N_4_. This type-II heterojunction exhibits better CO_2_ reduction performance than a single photocatalyst (Figure 2a). Although a type-Ⅱ heterojunction can inhibit the recombination of electrons and holes through charge space separation, the electrons gather in the CB of NiAl-LDHs, and the holes gather in the VB of carbon nitride. Thermodynamically, this charge transfer mechanism leads to an overall decrease in the redox ability of the composite photocatalyst. The Z-scheme photocatalysts can achieve both the spatial separation of the charges and maintain the strong redox capabilities of the photocatalyst [55,56,57]. Zhu et al. constructed a g-C_3_N_4_/rGO/PDIP Z-scheme photocatalyst using rGO as an electron conductor [58]. A fast electron transfer channel is formed between g-C_3_N_4_ and PDIP, and electrons from the CB of PDIP are migrated to the VB of carbon nitride through rGO under illumination. Yong et al. successfully prepared hollow Pt/g-C_3_N_4_/TiO_2_/IrOx Z-scheme photocatalysts via loading g-C_3_N_4_ onto TiO_2_ hollow spheres and modifying the co-catalyst [59]. Photogenerated electrons in TiO_2_ move towards g-C_3_N_4_, driven by the built-in electric field (Figure 2b). This Z-scheme without electron transfer intermediates has the advantage of a simple preparation and strong redox capacity. However, a direct Z-scheme heterojunction system is not well explained in terms of fermi level matching, internal electric field construction, and the driving factors of a charge transfer. To address the current shortcomings in Z-scheme heterojunction photocatalysts, Yu et al. analyzed the problems of Z-scheme heterojunctions and further put forward the concept of step-scheme (S-scheme) heterojunctions and systematically explained their photocatalytic mechanism (Figure 2c) [38,46]. Since then, the S-scheme photocatalyst has received increasing attention.

In the past three years, the development of carbon nitride-based S-scheme photocatalysts has been flourished, which has raised the wave of interests and reached a crescendo in the field of energy catalysis. Therefore, a timely overview on the recent progress of carbon nitride-based S-scheme heterojunctions is highly desirable, not only to unveil the basic working mechanism, but also to inspire future research directions in carbon nitride-based heterojunctions. This review introduces the development of carbon nitride-based S-scheme photocatalysts; comprehensively discusses the design principles, preparation methods, characterization techniques, and performance enhancement mechanisms of carbon nitride-based S-scheme photocatalysts; and systematically summarizes the research progress of carbon nitride-based S-scheme photocatalysts in a photocatalytic CO_2_ reduction, H_2_ evolution, and other solar energy conversions. Finally, some challenges faced by carbon nitride-based S-scheme photocatalysts and their future development directions are analyzed in depth.

## 2. Preparation of Carbon Nitride-Based S-scheme Photocatalyst

### 2.1. Design Principles

**Figure 3:**
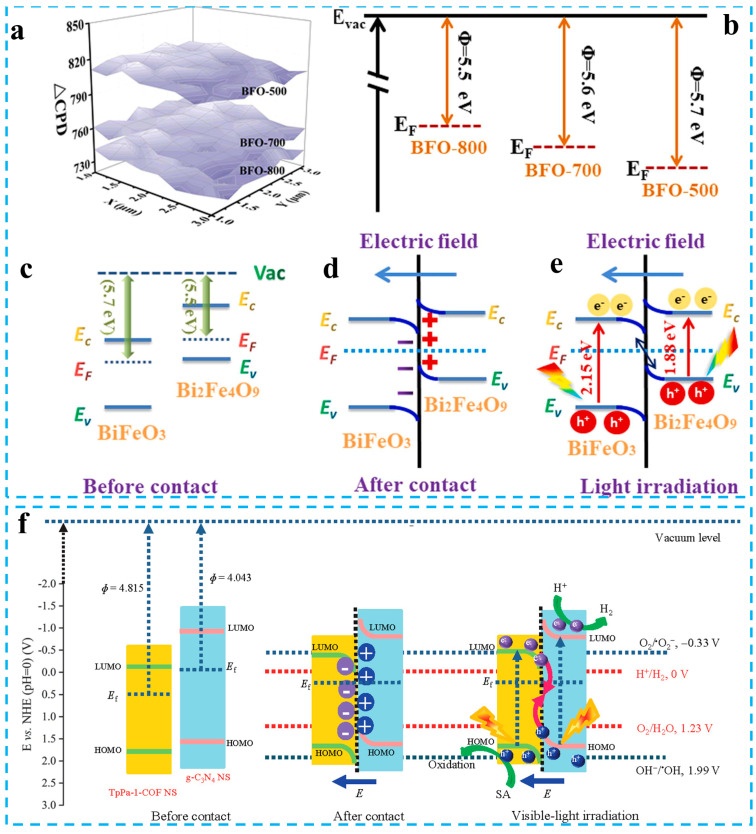
(**a**) Kelvin probes and (**b**) relative work functions of different catalysts, (**c**–**e**) interfacial charge transfer between BiFeO_3_ and Bi_2_Fe_4_O_9_. Reprinted with permission from ref. [60], Copyright 2020 Elsevier. (**f**) Schematic illustration of the formation mechanism and charge migration in the TPCNNS S-scheme heterojunction. Reprinted with permission from ref. [72], Copyright 2022 Wiley.

S-scheme heterojunction photocatalysts have similar charge transfer characteristics to direct Z-scheme heterojunctions, but there are some differences in design principles [60,61,62]. Different from the Z-scheme photocatalysts, which are constructed with n-type semiconductors, the S-scheme photocatalyst consists of an oxidation semiconductor photocatalyst (OP) and a reduction semiconductor photocatalyst (RP). Therefore, the S-scheme heterojunction photocatalysts can be an n–n junction, p–n junction, n–p junction, and p–p junction as long as the fermi level and band structure are appropriate [63,64,65]. In an S-scheme heterojunction photocatalyst, the OP needs to have a lower CB, VB, and fermi level than the OP, and the energy bands of the OP and RP are staggered (Figure 3a–c). The difference in fermi levels causes the spontaneous transfer of some electrons from the RP to the OP at the contact surface, and the uneven charge distribution induces the built-in electric field from the RP to the OP. The energy bands near the interface are also bent by electrostatic repulsion (Figure 3d). The carriers can transfer directionally under the built-in electric field, band bending, and coulomb force [66,67]. This transfer way of carrier will promote the recombination of holes and electrons with weak redox ability and prolong the lifetime of the carriers with strong redox capabilities. Therefore, S-scheme heterojunction photocatalysts have enhanced redox ability.

Pure carbon nitride is usually an n-type semiconductor, and there are three types of carbon nitride-based S-scheme photocatalysts: n–n, p–n, and n–p type [68,69,70]. The carbon nitride has quite a negative CB position, and the photogenerated electrons on it have strong reductive ability. Therefore, carbon nitride is typically used as an RP in S-scheme photocatalysts. Semiconductors whose CB, VB, and fermi levels are lower than carbon nitride can be used as the OP. When the carbon nitride-based S-scheme photocatalysts are excited by light, the electrons in the CB of the OP will recombine with the holes in the VB of carbon nitride under the coulomb force and built-in electric field (Figure 3e). The photogenerated electrons in carbon nitride and the photogenerated holes in the OP can fully exert their strong redox capabilities [71]. Zhang et al. prepared TpPa-1-COF/g-C_3_N_4_ S-scheme photocatalysts using a covalent organic framework and carbon nitride nanowires [72]. The TpPa-1-COF has a lower CB, VB, and fermi level than g-C_3_N_4_ nanowires (Figure 3f). The π-π conjugated heterointerface between the TpPa-1-COF and g-C_3_N_4_ has an enhanced interface electric field. The robust internal electric field makes the charge move quickly, which improves the charge separation and utilization of TpPa-1-COF/g-C_3_N_4_. Therefore, the TpPa-1-COF/g-C_3_N_4_ S-scheme heterojunction shows an excellent performance of photocatalytic hydrogen production (1153 μmol g^−1^ h^−1^). Chen et al. reported the IB/CNx S-scheme photocatalyst composed of iodine-doped BiOBr and nitrogen-deficient g-C_3_N_4_. The CB potential of IB is not negative enough, and the electrons on it have a low reduction ability. These electrons with a low reduction ability are driven by the internal electric field to recombine with the holes with a low oxidation ability on CNx. This charge transfer pathway leads to a prolonged lifetime of strong oxidizing holes on IB and strong reducing electrons on CNx. The IB/CNx S-scheme heterojunction has high-speed carrier migration and effective charge separation and exhibits enhanced photocatalytic activity in CO_2_ reduction and tetracycline degradation [73]. In addition, there are also some semiconductors whose CB, VB, and fermi level are higher than carbon nitride. At this time, carbon nitride can be used as the OP to construct S-scheme photocatalysts. However, the VB position of carbon nitride is not positive enough, and the oxidation capacity of this S-scheme heterojunction may be insufficient [74].

### 2.2. Preparation of Carbon Nitride-Based S-scheme Photocatalysts

**Figure 4:**
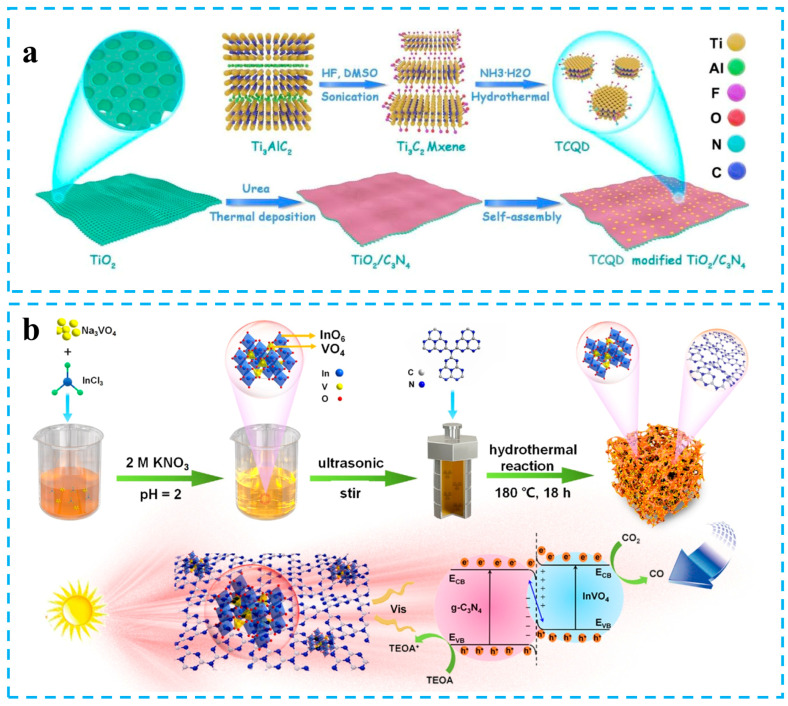
(**a**) Process diagram of the synthesis of TCQD-modified TiO_2_/C_3_N_4_. Reprinted with permission from ref. [78], Copyright 2020 Elsevier. (**b**) Schematic illustration of the synthesis of InVO_4_/CN. Reprinted with permission from ref. [79], Copyright 2021 Elsevier.

Carbon nitride is an organic polymer semiconductor that is easy to prepare and has stable physicochemical properties. There are many methods to construct S-scheme heterojunctions based on carbon nitride. The typical synthesis method of carbon nitride-based S-scheme photocatalysts includes coprecipitation, the high-temperature solid-state method, the hydrothermal method, the vapor deposition method, electrostatic self-assembly, the thermal polymerization method, etc. [75,76,77]. Macyk et al. prepared TiO_2_/C_3_N_4_/Ti_3_C_2_ composites with thermal polymerization and electrostatic self-assembly. The C_3_N_4_ layer is in situ grown on TiO_2_ nanosheets using urea as a precursor, and then, Ti_3_C_2_ quantum dots are loaded on a core-shell TiO_2_/C_3_N_4_ via electrostatic self-assembly (Figure 4a). TiO_2_/C_3_N_4_/Ti_3_C_2_ composite heterojunction photocatalysts follow the S-scheme charge transfer characteristics in CO_2_ reduction and exhibit enhanced CO and CH_4_ generation activity. The S-scheme charge transfer pathway between TiO_2_ and C_3_N_4_ is beneficial to inhibit the strong redox electron and hole recombination. Ti_3_C_2_ can promote electron transfer and increase the CO_2_ reduction rate [78]. Chen et al. prepared bulk carbon nitride via thermal polymerization using melamine as a precursor and then added bulk carbon nitride to the solution containing V source and In source. The InVO_4_ quantum dots/g-C_3_N_4_ S-scheme heterojunctions (InVO_4_/CN) were finally synthesized via ultrasonic and hydrothermal cutting methods (Figure 4b). The CO_2_ reduction rate of 0D/2D hybrid InVO4/CN S-scheme photocatalyst reached 69.8 μmol g^−1^ h^−1^, and the content of CO in the product was as high as 93.3%. The InVO_4_/CN obtained via this method had abundant defect sites, which were beneficial to the adsorption and activation of CO_2_ [79].

Cui et al. prepared anatase TiO_2_/H-doped rutile TiO_2_/g-C_3_N_4_ (TiCN) double S-scheme heterojunction photocatalysts with the electrostatic self-assembly method. First, g-C_3_N_4_ nanosheets, anatase TiO_2_ nanoparticles, and H-doped rutile TiO_2_ nanorods are prepared separately, and then, the three materials are spontaneously assembled with electrostatic force in an ethanol–water mixture. Anatase TiO_2_ nanoparticles and H-doped rutile TiO_2_ nanorods are loaded on g-C_3_N_4_ ultra-thin nanosheets. The photocatalytic hydrogen evolution rate of this double S-scheme heterojunction catalyst is 62.37 mmol g^−1^ h^−1^, and the quantum efficiency at 365 nm is 45.9%. The ternary TiCN photocatalyst has suitable lattice matching and fast charge transport channels, which is beneficial to the separation and transfer of photogenerated carriers [80]. Ye et al. prepared S-doped g-C_3_N_4_/N-doped MoS_2_ S-scheme photocatalysts (NMS/SCN) using thiourea and copper chelator as raw materials via the thermal polymerization method. The S released by thiourea during calcination is doped in g-C_3_N_4_, and the N released by copper chelating agent is doped in MoS_2_. The charge transfer between NMS and SCN follows the S-scheme. The electrons and holes are distributed on SCN and NMS, respectively, and the carrier recombination is effectively suppressed. The hydrogen evolution rate of NMS/SCN photocatalysis is 658.5 μmol g^−1^ h^−1^, which is 23 and 38 times that of NMS and SCN, respectively [81]. Different methods were used for preparing carbon nitride-based S-scheme photocatalysts, as shown in Table 1. In summary, the construction of an S-scheme photocatalyst is a simple and efficient way to enhance the photocatalytic performance of carbon nitride. In addition, defect engineering, interfacial engineering, and co-catalyst modification can be combined with the S-scheme heterojunction to promote the performance of carbon nitride-based heterojunction photocatalysts.

### 2.3. Techniques for the Identification of Carbon Nitride-Based S-scheme Photocatalysts

**Figure 5:**
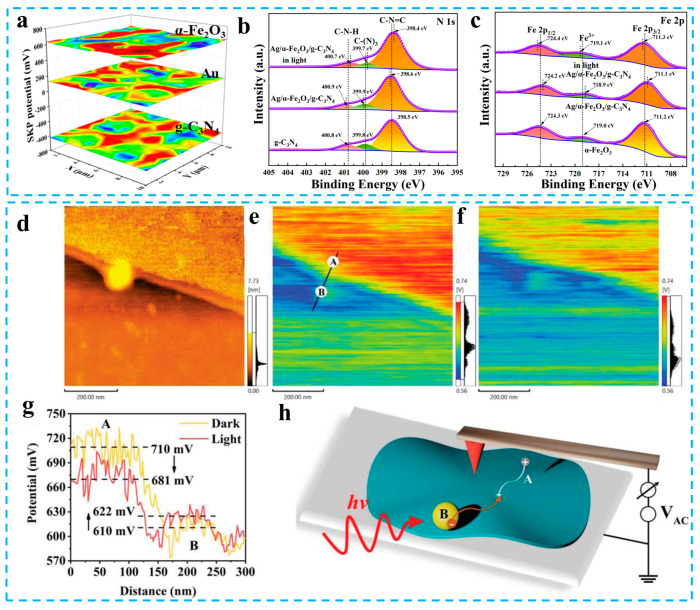
(**a**) SKP map of Au, α-Fe_2_O_3,_ and g-C_3_N_4_. XPS spectra of (**b**) N 1s and (**c**) Fe 2p. Reprinted with permission from ref. [83]. (**d**) Topographic map of catalyst. The surface potential distribution of the catalyst under (**e**) darkness and (**f**) light. (**g**) The trend of potential between A and B. (**h**) Sketch of KPFM test device. Reprinted with permission from ref. [82], Copyright 2021 Wiley.

With the progress of characterization technology and the continuous exploration of researchers, the carrier transfer characteristics of an S-scheme photocatalyst has been gradually verified. An in situ irradiation X-ray photoelectron spectroscopy (ISIXPS) and kelvin probe force microscope (KPFM) are two characterization techniques that can directly prove the electronic transfer in S-scheme photocatalysts. XPS is a high-precision instrument for detecting the elemental and valence of solid materials. The binding energy in the spectrum is related to the change in electron density. After the element loses some electrons, its electron density decreases. Therefore, the binding energy of the elements that lose electrons will increase; on the contrary, the binding energies of the elements that get electrons will decrease. The changing trend of the binding energy of different elements can prove the formation of the interface electric field of an S-scheme photocatalyst and the direction of electron transfer under irradiation. For example, in the S-scheme photocatalyst constructed by α-Fe_2_O_3_ and g-C_3_N_4_, g-C_3_N_4_ has a smaller work function than α-Fe_2_O_3_ (Figure 5a). After contact, the electrons of carbon nitride transfer spontaneously to α-Fe_2_O_3_. The loss of electrons in g-C_3_N_4_ results in a positive shift in the binding energy of N and a decrease in the binding energy of Fe in α-Fe_2_O_3_ after obtaining electrons (Figure 5b,c). The inhomogeneous charge distribution forms an interfacial electric field from g-C_3_N_4_ to α-Fe_2_O_3_. When the composite photocatalyst is excited by light, the photogenerated electrons of α-Fe_2_O_3_ move to carbon nitride under the action of the interfacial electric field. At this time, g-C_3_N_4_ loses electrons and α-Fe_2_O_3_ gets electrons, so the binding energy of N and Fe shift positively and negatively, respectively [83]. Therefore, ISIXPS can characterize the direction of electron transfer in S-scheme heterojunctions. KPFM can draw a topographic map and potential distribution map of the sample surface. Yu et al. used KPFM to study charge transfer characteristics in S-scheme photocatalysts composed of CdS and pyrene-based conjugated polymers (PT) (Figure 5h). The PT has a smaller work function than CdS according to the contact potential difference between pure CdS and pure PT. The distribution of the charge on CdS/PT in the dark is shown in Figure 5e. The electrons on PT are transferred to CdS and its surface is positively charged, so the surface potential of PT is higher compared to the CdS. The potential difference between CdS and PT indicates the existence of an interfacial electric field. The surface potential of PT decreases and the potential of CdS increases under light, indicating that photogenerated electrons are moved from CdS to PT (Figure 5f,g) [82]. Therefore, KPFM can also visually characterize the electron transfer in S-scheme photocatalysts.

**Figure 6:**
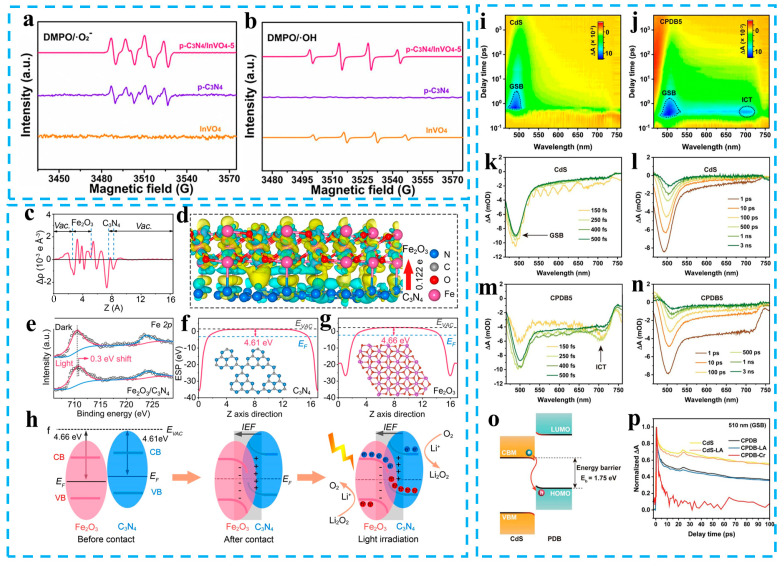
(**a**) O_2_^−^ and (**b**)·OH signal of photocatalysts. Reprinted with permission from ref. [85], Copyright 2022 Elsevier. The average electron density difference (**c**) and differential charge density map (**d**) of the heterojunction photocatalyst. (**e**) In situ XPS spectra of Fe 2p. The work functions of C_3_N_4_ (**f**) and Fe_2_O_3_ (**g**). (**h**) The charge transfer process between Fe_2_O_3_ and C_3_N_4_. Reprinted with permission from ref. [86], Copyright 2022 Wiley. TA spectra of (**i**,**k**,**l**) CdS and (**j**,**m**,**n**) CPDB5. (**o**) The charge transfer pathways between CdS and PDB. (**p**) Normalized kinetic curves of CdS and CPDB5. Reprinted with permission from ref. [87], Copyright 2023 Wiley.

Other techniques can also indirectly prove or assist in proving the formation of S-scheme heterojunctions, such as the scanning kelvin probe (SKP), surface photovoltage (SPV), femtosecond transient absorption (fs-TA), and electron paramagnetic resonance (EPR). An SKP can test the work function of a semiconductor to get information about the fermi level and provide a basis for the identification of the interface electric fields in heterojunctions. Similar characterization technologies also include ultraviolet photoelectron spectroscopy (UPS) [84]. The SPV signal can provide feedback information about charge transfer, which is similar to a transient photocurrent, electrochemical impedance spectroscopy (EIS), photoluminescence (PL), etc. The built-in electric field can drive the rapid transfer of charge carriers to form a fast and strong SPV response. The peaks of OH and O_2_^−^ in the EPR spectrum are related to the redox ability of photogenerated carriers in the photocatalyst. A different electronic transfer mechanism can lead to changes in the redox property of the heterojunction photocatalyst. The charge transfer characteristics in the photocatalyst can be judged by the trend of the radical signal. Hu et al. verified the charge transfer characteristics of g-C_3_N_4_/InVO_4_ S-scheme photocatalysts by EPR [85]. The single carbon nitride is not sufficient to oxidize water to produce ·OH, and the single InVO_4_ is not sufficient to reduce oxygen to produce ·O_2_^−^. The ERP spectrum of the g-C_3_N_4_/InVO_4_ composite photocatalyst shows significantly enhanced signal peaks for ·O_2_^−^ and OH, indicating that the charge transfer characteristics in g-C_3_N_4_/InVO_4_ is an S-scheme rather than type-II (Figure 6a,b). The interface charge transfer characteristics of S-scheme photocatalysts can also be verified with DFT calculations, including the average planar electron density difference Δρ(z) and differential charge density map. Li et al. verified the charge transfer pathways of Fe_2_O_3_/C_3_N_4_ S-scheme heterojunction via DFT calculations [86]. The average planar electron density curve and differential charge density map show that the electrons move from C_3_N_4_ to Fe_2_O_3_, resulting in the interfacial electric field being created from C_3_N_4_ to Fe_2_O_3_ (Figure 6c,d). Therefore, DFT calculations can provide a basis for judging the formation of the interface electric field. Fs-TA can simulate the quenching path and corresponding lifetime of carriers in semiconductors by extracting spectral attenuation features. Yu et al. used the fs-TA technique to study the photophysical processes of S-scheme heterojunctions such as cadmium sulfide/pyrene-ALT-difluorobenzothiadiazole (CdS/PBD) and detected the charge transfer signal of the S-scheme and the lifetime of this transfer process [87]. Figure 6m shows an additional peak near 710 nm, the energy corresponding to its wavelength approximately equal to the energy released by the S-scheme charge transfer between CdS and PDB (Figure 6o). Therefore, it is considered to be the direct evidence for the formation of the S-scheme heterojunction between CdS and PDB. 

A built-in electric field-driven S-scheme charge transfer can efficiently separate electrons and holes. Therefore, photoelectrochemical tests and photoluminescence spectra can also demonstrate the successful construction of S-scheme heterojunctions to some extent. The transfer of photogenerated electrons along the external circuit will generate a photocurrent. The higher the separation efficiency of photogenerated electrons and holes, the greater the photocurrent generated [84,85]. The transient photocurrent response of p-C_3_N_4_/InVO_4_ is significantly higher than that of the original p-C_3_N_4_ and InVO_4_, indicating that p-C_3_N_4_/InVO4 has higher charge separation efficiency, which means that more electrons and holes can participate in the photocatalytic reaction [85]. The EIS can also prove that the S-scheme heterojunction has excellent charge transfer characteristics. In the LaVO_4_/g-C_3_N_4_ S-scheme heterojunction system, the composite has the smallest arc radius and the best photocatalytic performance. The original g-C_3_N_4_ has the largest arc radius and the worst photocatalytic activity, indicating that the S-scheme heterojunction is beneficial to reduce the charge transfer resistance and promote the carrier separation [84]. The PL spectrum can represent the intensity of electron and hole recombination luminescence. Stronger PL peaks mean higher electron-hole complexation rates. The separation efficiency of electrons and holes can also be analyzed via a fluorescence lifetime. The fluorescence lifetime is equivalent to the existence time of photogenerated carriers. The longer the fluorescence lifetime of the photocatalyst means the better the separation of electrons and holes [80,84].

## 3. Application of Carbon Nitride-Based S-scheme Photocatalysts in Solar Energy Conversion

Since it was first discovered to have catalytic activity in photocatalytic H_2_ evolution, carbon nitride has been widely used as a carrier for various photocatalytic reactions [88,89,90]. The carbon nitride-based S-scheme photocatalysts have enhanced carrier separation and migration capabilities. The further enhancement of the redox capacity allows carbon nitride-based S-scheme photocatalysts to show satisfactory activity in photocatalytic solar energy conversion.

### 3.1. Photocatalytic H_2_ Evolution 

H_2_ is a kind of energy with significant advantages. It is an effective way to alleviate the energy shortage by replacing fossil energy with H_2_. Photocatalytic water splitting is a green H_2_ production technology, which is expected to be promoted on a large scale. The development of high-performance semiconductor photocatalysts is crucial to achieve this goal. The direct decomposition of H_2_O to produce H_2_ and O_2_ requires a high energy barrier of 237 KJ mol^−1^, and the band structure of photocatalysts needs to match the redox potential of water [91]. The suitable photocatalyst should have more negative CB than H_2_ reduction potential (pH = 0, 0 V vs. NHE) and more positive VB than H_2_O oxidation potential (pH = 0, 1.23 eV vs. NHE). In addition to the basic thermodynamic requirements, hydrogen or oxygen evolution reactions on the surface of the catalyst will require more energy due to the overpotential [92]. Carbon nitride is a kind of polymer semiconductor suitable for photocatalytic hydrogen production because its CB potential is negative enough. The unique carrier transfer mechanism of S-scheme photocatalyst makes the redox ability of the OP and RP complement each other. Therefore, the S-scheme photocatalysts constructed by carbon nitride and the OP possess a strong redox capacity. The electrons from the OP are recombined with holes on carbon nitride, the holes from the OP are used to oxidize H_2_O to produce oxygen, and the electrons of carbon nitride can fully participate in the hydrogen evolution reaction.

**Figure 7:**
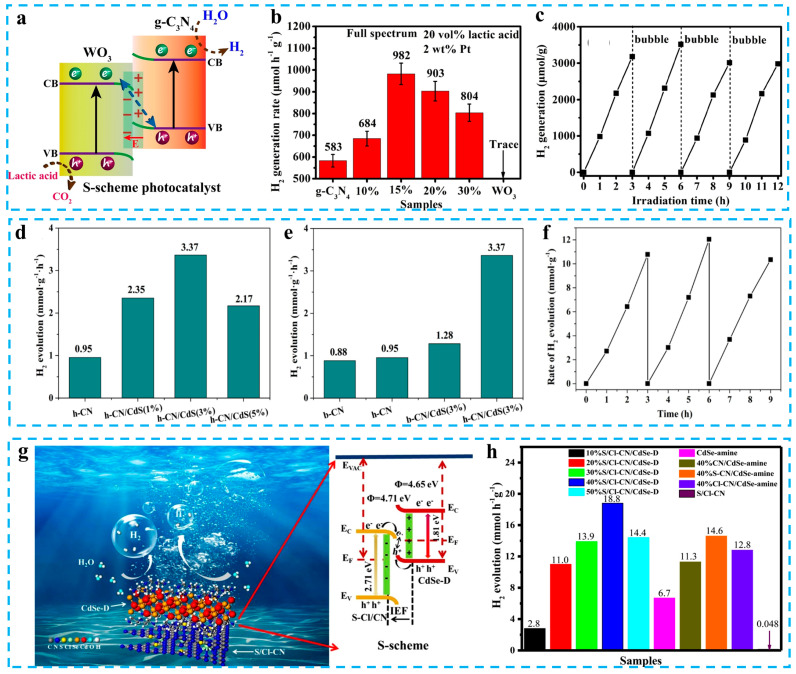
(**a**) Photocatalytic mechanism of WO_3_/g-C_3_N_4_ S-scheme heterojunction. (**b**) H_2_ evolution activity of different samples. (**c**) Recycling test of 15% WO_3_/g-C_3_N_4_. Reprinted with permission from ref. [93], Copyright 2019 Elsevier. (**d**,**e**) The photocatalytic H_2_ generation of different photocatalysts. (**f**) Recycling test for hydrogen evolution of h-CN/CdS (3%). Reprinted with permission from ref. [94], Copyright 2022 Elsevier. (**g**) Schematic illustration of the photocatalytic mechanism of AS-Cl/CN/CdSe-D. (**h**) H_2_ evolution of different photocatalysts. Reprinted with permission from ref. [95], Copyright 2021 Elsevier.

An S-scheme heterojunction can usually be combined with doping, defect, and morphology engineering to further improve the activity of photocatalysts. Yu et al. obtained carbon nitride nanowires via two-step pyrolysis, and then, WO_3_/g-C_3_N_4_ 2D/2D S-scheme photocatalysts were synthesized via electrostatic self-assembly with WO_3_ nanowires. The photocatalytic H_2_ evolution rate of the optimal sample is 982 μmol h^−1^ g^−1^, which is 1.7 times that of the original g-C_3_N_4_ nanosheet (Figure 7b). In addition, this composite photocatalyst exhibited satisfactory stability in cycling experiments (Figure 7c). XPS, EPR, and DFT calculations are used to demonstrate the formation of an interfacial electric field in the vicinity of the contact surface between WO_3_ and g-C_3_N_4_. The enhanced photocatalytic activity of WO_3_/g-C_3_N_4_ is mainly attributed to the interfacial electric field accelerating charge separation (Figure 7a). The electrons in WO_3_/g-C_3_N_4_ are retained on the CB of g-C_3_N_4_ under the effect of the built-in electric field, which in turn reduces the hydrogen protons to H_2_ [93]. An et al. constructed an h-CN/CdS S-scheme heterojunction via self-assembly. The hydrogen precipitation rate of the best ratio h-CN/CdS photocatalyst is 3.37 mmol g^−1^ h^−1^, which is greatly improved compared to the original sample (Figure 7d,e). This S-scheme heterojunction accelerates the charge separation, inhibits the photo-corrosion of CdS, and keeps h-CN/CdS stable in the photocatalytic reaction (Figure 7f) [94]. Chen et al. successfully synthesized S/Cl-CN/CdSe-D S-scheme heterojunction photocatalysts via growing CdSe-amine on the surface of carbon nitride co-doped with sulfur and chlorine (Figure 7g). The H_2_ evolution rate of S/Cl-CN/CdSe-D is 18.8 mmol g^−1^ h^−1^, which is 391 and 2.8 times that of S/Cl-CN and CdSe-D, respectively. The apparent quantum efficiency of S/Cl-CN/CdSe-D at 420 nm is 38.4% (Figure 7h). The high hydrogen production activity is related to the built-in electric field accelerating the migration of photogenerated carriers. The interfacial electric field suppresses the loss of electrons on CdSe-D so that more electrons are involved in the proton reduction discharge, improving the utilization of photogenerated carriers [95]. More recent examples of carbon nitride-based S-scheme photocatalysts’ photocatalytic water splitting are shown in Table 2 [96,97,98,99,100,101,102,103,104,105,106].

### 3.2. Photocatalytic CO_2_ Reduction

Excessive consumption of fossil fuels has led to the gradual increase of CO_2_ in the atmosphere, causing a chain of global issues, such as the greenhouse effect and an abnormal climate [107]. Plants in nature are able to fix CO_2_ and release O_2_ through photosynthesis to maintain the carbon/oxygen cycle in ecosystems. Semiconductor photocatalysis can mimic the photosynthesis of plants by using sunlight as the sole source of energy to drive the conversion of H_2_O and CO_2_ into high value-added products. This technology solves the problem of solar energy storage and enables CO_2_ reduction [108,109,110]. The CO_2_ molecule is quite thermodynamically stable, and its reduction reaction involves a multi-electron transfer process [111]. Photocatalytic CO_2_ reduction is more challenging than photocatalytic water splitting. The carbon nitride-based S-scheme photocatalysts have strong redox capabilities, which can drive a CO_2_ reduction reaction with a high thermodynamic barrier. The built-in electric field in the S-scheme photocatalyst can accelerate the photogenerated charge transfer and promote the photocatalytic reaction rate. Therefore, carbon nitride-based S-scheme photocatalysts have been extensively studied in CO_2_ reduction.

**Figure 8:**
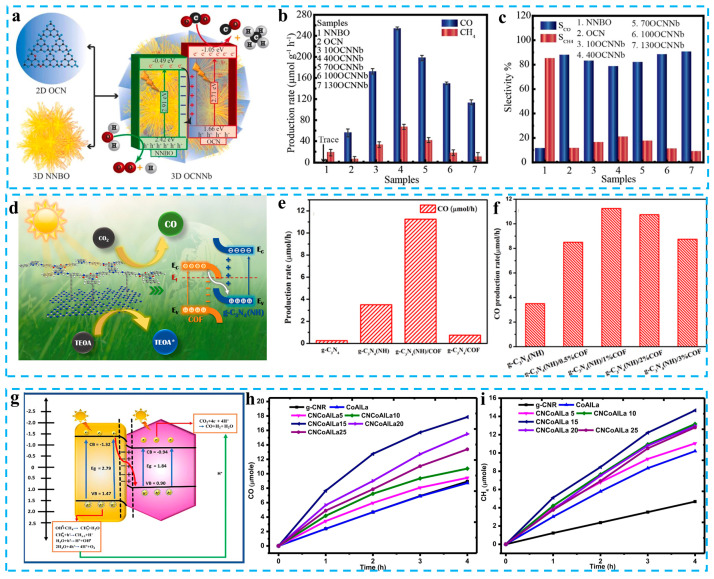
(**a**) Mechanism of photoreduction of CO_2_ over OCNNb. (**b**) Products rate of CO and CH_4_ of different photocatalysts. (**c**) Cycling test of CO_2_ photoreduction over 40OCNNb. Reprinted with permission from ref. [112], Copyright 2022 Elsevier. (**d**) The charge transfer mode of composite photocatalyst. (**e**) The CO_2_ reduction rate of the different photocatalysts. (**f**) Yields of CO for g-C_3_N_4_ (NH)/COF with different amounts of COF. Reprinted with permission from ref. [113], Copyright 2022 Elsevier. (**g**) Schematic representation of charge transfer in g-CNR/CoAlLa-LDH. (**h**,**i**) Yields of CO and CH_4_ for each photocatalyst. Reprinted with permission from ref. [114], Copyright 2022 Elsevier.

The design of hierarchical composite photocatalysts is one of the important methods to promote the photoreduction of CO_2_ into fuel. Xiu et al. reported a layered S-scheme heterojunction (OCN/NNBO) assembled with N-doped Nb_2_O_5_ and O-doped carbon nitride (Figure 8a). The photocatalytic r yields rates of CO_2_ to CH_4_ and CO are 68.11 and 253.34 μmol g^−1^ h^−1^, respectively (Figure 8b,c). The layered S-scheme heterostructure accelerates the migration of carriers and provides a large specific surface area and abundant active sites, which improves the photocatalytic performance of OCN/NNBO. The S-scheme charge transfer leaves electrons on the CB of the OCN, and these strongly reducing electrons react with the CO_2_ adsorbed on the surface to reduce CO_2_ to CO and CH_4_; isotope experiments also show that CO_2_ is the only source of carbon for the production of CO and CH_4_ [112]. The composites composed of carbon nitride and a covalent organic framework (COF) have significant advantages in improving interfacial contact and accelerating the charge transfer. Ye et al. coupled g-C_3_N_4_ and Tp-TtaCOF with nitrogen defects via evaporation-induced self-assembly and successfully prepared a 2D/2D C_3_N_4_ (NH)/COFS heterojunction photocatalyst (Figure 8d). The reduction rate of CO_2_ to CO by C_3_N_4_ (NH)/COF is 11.25 μmol h^−1^, which is 45 times that of the original carbon nitride, and the selectivity is as high as 90.4% (Figure 8e,f). The experiments and DFT calculations show that C_3_N_4_ (NH)/COF has proper nitrogen-vacancy and a robust internal electric field, which is beneficial for CO_2_ trapping, activation, and charge separation. Under the S-scheme charge transfer mechanism, electrons are gathered on the CB of g-C_3_N_4_ and gradually reduce the CO_2_ adsorbed on the surface to produce intermediates such as CO_2_*, COOH*, and CO*, which are further reduced to produce CO [113]. Mohamed et al. prepared g-CNR/CoAlLa-LDH binary photocatalysts via combining 0D carbon nitride nanorods with 2D trimetal CoAlLa LDH nanowires (Figure 8g). The photocatalytic yields rates of CH_4_ and CO were 36.66 and 44.62 μmol g^−1^ h^−1^, respectively (Figure 8h,i). The charge transfer between g-CNR and CoAlLa-LDH follows the S-scheme, and the electrons and holes are left on different semiconductors, which effectively promote the separation of photogenerated carriers [114]. More recent cases of the photocatalytic CO_2_ reduction of carbon nitride-based S-scheme photocatalysts are shown in Table 3 [115,116,117,118,119,120,121,122,123,124,125].

### 3.3. Other Applications of Carbon Nitride-Based S-scheme Photocatalysts

Besides the two typical photocatalytic solar energy conversions of photocatalytic H_2_ generation and CO_2_ reduction, carbon nitride-based S-scheme photocatalysts have also attracted much attention in the fields of H_2_O_2_ production, environmental purification, antibacterial properties, etc. H_2_O_2_ is a high-value-added chemical known as a green molecule, which is widely used in medical, chemical, and other fields. Photocatalytic H_2_O_2_ production has the advantage of environmental friendliness and a low cost. Wang et al. reported an S-scheme photocatalyst composed of ultrathin carbon nitride and polydopamine with an H_2_O_2_ yield of 3801 μmol g^−1^ h^−1^, which is 2 times and 11 times that of the original carbon nitride and polydopamine, respectively. The enhanced activity of the composite photocatalysts is due to the accelerated charge transfer by S-scheme heterojunctions. Experiments show that H_2_O_2_ is generated in the presence of O_2_, so H_2_O_2_ may be derived from an O_2_ reduction reaction rather than an H_2_O oxidation reaction. In addition, H_2_O_2_ production was inhibited under the condition of scavenging ·O_2_^−^, so H_2_O_2_ was derived from a two-step single-electron O_2_ reduction reaction [126]. Li et al. synthesized an iron-tungsten oxide-modified, oxygen-rich carbon nitride with defects (ITOs/OCNv) S-scheme heterojunction composites. The introduction of double defects expands the spectral absorption, and the S-scheme charge transfer pathway promotes the separation of carriers. The degradation rates of sulfamethazine and ciprofloxacin by ITOs/OCNv within 40 minutes are 99.6% and 99.5%, respectively, and the ITOs/OCNv shows excellent resistance to water quality changes. Experiments show that the internal electric field in ITOs/OCNv can promote carrier separation, and more photogenerated electrons can effectively participate in O_2_ reduction and persulfate activation, resulting in a large number of active free radicals to remove pollutants [127]. Zhang et al obtained carbon nitride nanowires via two-step annealing. The carbon nitride nanowires were added to an N-dimethylformamide solution containing cerium nitrate, and CeO_2_ quantum dots/carbon nitride nanowires S-scheme photocatalysts were obtained via oil bath and calcination. The photocatalytic sterilization rate of CeO_2_/g-C_3_N_4_ to staphylococcus aureus was as high as 88.1% under visible light. It can be attributed to the rapid charge separation of S-scheme photocatalysts and produced more sterilized active substances [128].

## 4. Conclusions and Outlook

In summary, photocatalysis has been extensively studied as a technology to realize solar energy conversion. The key to advances in photocatalysis is the development of highly efficient photocatalysts. The design of carbon nitride-based S-scheme photocatalysts as a promising strategy to achieve efficient solar energy conversion has been widely studied. In this review, we systematically review the design strategy of an S-scheme based on carbon nitride and its engineering application in energy conversion, and we summarize the progress of a carbon nitride-based S-scheme heterojunction from the following aspects: (i) the design of S-scheme heterojunction based on carbon nitride, (ii) the method for the preparation of carbon nitride-based S-scheme photocatalysts, (iii) the characterization of built-in electric field and charge transfer characteristics, (iv) the photocatalytic mechanism of carbon nitride-based S-scheme photocatalysts, and (v) the application of a carbon nitride-based S-scheme catalyst in a photocatalytic solar energy conversion.

Some challenges and suggestions are also raised in this work. The carbon nitride-based S-scheme photocatalyst has robust redox capabilities, rapid charge dynamics, and can cooperate with doping, defect engineering, monoatomic modification, precious metal modification, and other measures to enhance the photocatalytic performance. However, there are still some unsatisfactory aspects in the design, preparation, and mechanism analysis of carbon nitride-based S-scheme photocatalysts. In order to further develop the carbon nitride-based S-scheme photocatalyst and promote its practical application in photocatalytic solar energy conversion, the following notable problems and future development opportunities are put forward on the basis of the current research:

1. The carbon nitride has a rather negative CB potential, so carbon nitride generally acts as a reduction photocatalyst matched to oxidation photocatalysts with a wide band gap to construct S-scheme heterojunctions. However, this design scheme is not conducive to improving the spectral response of heterojunction photocatalysts. The band structure of carbon nitride can be continuously and controllably adjusted via doping engineering [129,130]. The carbon nitride is expected to be used as oxidation semiconductors to construct broad-spectrum response S-scheme heterojunctions with narrow bandgap semiconductors such as Bi_2_S_3_ and CuInS_2_;
2. The preparation method of a carbon nitride-based S-scheme photocatalyst often requires complex steps, so it is difficult to achieve large-scale production. In addition, conventional assembly methods, such as electrostatic self-assembly/physical adsorption, may also suffer from inadequate contact between the two components. Therefore, the simple one-step method or in situ growth process needs to be further studied. It is also necessary to expand the contact interface of the heterojunction, simplify the processes, and reduce the preparation cost of the photocatalyst;
3. The related characterization techniques of S-scheme heterojunctions still need to be further developed. The built-in electric field is the key driver for rapid charge separation in S-scheme photocatalysts, but conventional characterization methods make it difficult to accurately characterize the strength of the internal electric field and its position relative to the active center. Li et al. used time-resolved photoelectron microscopy and surface photovoltage microscopy to directly observe the charge transfer characteristics in nanoseconds. [131,132] These advanced in situ characterization techniques are expected to further reveal the action mechanism of an internal electric field in a carbon nitride-based S-scheme photocatalyst;
4. Photocatalytic solar energy conversion is a complex process. The carbon nitride-based S-scheme photocatalyst system usually includes defect engineering, surface plasmon resonance, photothermal effect, and so on. The synergistic mechanism of different factors needs to be further explored in depth;
5. The carbon nitride-based S-scheme photocatalyst has many advantages in solar energy conversion. However, there is still a big gap between its catalytic efficiency and the requirements of commercial application. Therefore, it is necessary to explore more efficient improvements of carbon nitride-based S-scheme photocatalysts, such as photo-electric synergy, photo-thermal synergy, and photo-magnetic synergy, in order to substantially enhance the catalytic efficiency of carbon nitride-based S-scheme photocatalysts.

## Figures and Tables

**Table 1:** Comparison of different preparation strategies of carbon nitride-based S-scheme photocatalysts.

Photocatalysts	Synthesis Strategy	Characteristics	Ref.
S-g-C_3_N_4_/TiO_2_	Electrospinning	Small diameter, uniform morphology, time and cost-consuming	[68]
CuFe_2_O_4_/Bi_2_WO_6_/mpg-C_3_N_4_	Hydrothermal and solvothermal	High crystallinity, controllable morphology, low efficiency	[69]
WO_3_/g-C_3_N_4_	Thermal polycondensation	Facile, highly efficient, uncontrollable morphology	[70]
TpPa-1-COF/g-C_3_N_4_	Self-assembly	Uniform morphology, complicated preparation process	[72]
70IB/CNx	Deposition-precipitation	Facile, highly efficient, easy aggregation	[73]
us-Cu_3_P|S/CN	Solid-state method	High-efficiency, simple, uncontrollable morphology	[74]
V_2_O_5_/C_3_N_4_	Mechanical agitation	Facile, highly efficient, relatively poor interface contacts	[76]
TiO_2_/C_3_N_4_/Ti_3_C_2_	Electrostatic self-assembly	High-efficiency, and low-cost, complex conditions	[78]
CdS/PT	In-situ growth	Uniform morphology, close contact, complicated preparation process	[82]

**Table 2:** Various carbon nitride-based S-scheme photocatalysts for H_2_ evolution reported in recent years.

Photocatalysts	Auxiliary Conditions	Light Source	H_2_ Evolution (μmol h^−1^ g^−1^)	Ref.
S-pCN/WO_2.72_	TEOA	Xe lamp (300 W, λ > 420 nm)	786.0	[96]
g-C_3_N_4_/Zn_0.2_Cd_0.8_S-DETA	Na_2_S, Na_2_SO_3_, H_2_PtCl_6_	Xe lamp (300 W, λ > 420 nm)	6690.0	[97]
g-C_3_N_4_/TiO_2_	TEOA, Pt	Xe lamp (300 W, λ > 420 nm)	974.6	[98]
BiOBr/g-C_3_N_4_	TEOA	Xe lamp (300 W)	106.63	[99]
ZnCo_2_S_4_/g-C_3_N_4_	TEOA	Xe lamp (300 W)	6619.0	[100]
ISCNMT	MeOH, K_2_PtCl_6_	Xe lamp (110 mW cm^−2^, λ > 400 nm)	9640.0	[101]
ZnIn_2_S_4_/g-C_3_N_4_/Ti_3_C_2_	TEOA	Xe lamp (300W, λ > 420 nm)	2452.1	[102]
TiO_2_-O_V_/g-C_3_N_4_	TEOA, H_2_PtCl_6_	Xe lamp (300 w, λ > 400 nm)	6308.0	[103]
S-g-C_3_N_4_-E	TEOA, Pt	Xe lamp (300 W, λ > 420 nm)	5548.1	[104]
ZnIn_2_S_4_/CD/g-C_3_N_4_	TEOA, NaCl, H_2_PtCl_6_	Xe lamp (300 W, λ > 420 nm)	17580	[105]
In_2.77_S_4_/NiS_2_/g-C_3_N_4_	TEOA	Xe lamp (300 W)	7481.7	[106]

**Table 3:** Recent cases of photocatalytic CO_2_ reduction of carbon nitride-based S-scheme photocatalysts.

Photocatalysts	Light Source	CH_4_ (μmol h^−1^ g^−1^)	CO (μmol h^−1^ g^−1^)	Ref.
g-C_3_N_4_/Bi/BiVO_4_	Xe lamp (300 W, λ > 420 nm)	-	1.25	[115]
CoO/PCN	Xe lamp (300 W, λ > 410 nm)	-	40.31	[116]
TiO_2_/Ti_3_AlC_2_/g-C_3_N_4_	HID car lamp (35 W)	2103.50	297.26	[117]
g-C_3_N_4_/CdSe-DETA	Xe lamp (300 W, λ > 420 nm)	-	25.87	[118]
g-C_3_N_4_/Fe-MOF	Xe lamp (300 W, full spectrum)	-	19.17	[119]
Bi_3_NbO_7_/g-C_3_N_4_	Xe lamp (300 W, λ > 420 nm)	37.60	4.18	[120]
CsPbBr_3_/S-g-C_3_N_4_	Xe lamp (300 W, λ > 400 nm)	-	83.60	[121]
ZnIn_2_S_4_/g-C_3_N_4_	Xe lamp (300 W, full spectrum)	-	883.00	[122]
Nb_2_O_5_/g-C_3_N_4_	Xe lamp (300 W, full spectrum)	20.89	171.98	[123]
BiOI/g-C_3_N_4_	Xe lamp (300 W, λ > 400 nm)	-	3.11	[124]
g-C_3_N_4_/Cu_2_O@Cu	Xe lamp (300 W, full spectrum)	3.10	10.80	[125]

## Data Availability

The data that support the findings of this study are available from the corresponding author upon reasonable request.
